# Global and local community memberships for estimating spreading capability of nodes in social networks

**DOI:** 10.1007/s41109-021-00421-3

**Published:** 2021-11-02

**Authors:** Simon Krukowski, Tobias Hecking

**Affiliations:** 1 Department of Computer Science and Applied Cognitive Science, University of Duisburg-Essen, Duisburg, Germany; 2 Institute for Software Technology, German Aerospace Center (DLR), Cologne, Germany

**Keywords:** Spreading, Networks, Link clustering, Community structure

## Abstract

The analysis of spreading processes within complex networks can offer many important insights for the application in contexts such as epidemics, information dissemination or rumours. Particularly, structural factors of the network which either contribute or hinder the spreading are of interest, as they can be used to control or predict such processes. In social networks, the community structure is especially relevant, as actors usually participate in different densely connected social groups which emerge from various contexts, potentially allowing them to inject the spreading process into many different communities quickly. This paper extends our recent findings on the community membership of nodes and how it can be used to predict their individual spreading capability (Krukowski and Hecking, in: Benito, Cherifi, Cherifi, Moro, Rocha, Sales-Pardo (eds) Complex networks & their applications IX. Springer, Cham, pp 408–419, 2021) by further evaluating it on additional networks (both real-world networks and artificially generated networks), while additionally introducing a new local measure to identify influential spreaders that—in contrast to most other measures, does not rely on knowledge of the global network structure. The results confirm our recent findings, showing that the community membership of nodes can be used as a predictor for their spreading capability, while also showing that especially the local measure proves to be a good predictor, effectively outperforming the global measure in many cases. The results are discussed with regard to real-world use cases, where knowledge of the global structure is often not given, yet a prediction regarding the spreading capability highly desired (e.g., contact-tracing apps).

## Introduction

The study of spreading processes on networks has a long research history in information diffusion in social networks (Guille et al. 2013), computer communication (Balthrop et al. 2004), and epidemiology (Nowzari et al. 2016). Insights in this regard are of high relevance for better control of spreading either for increasing influence and reach in social networks (Kempe et al. 2003) or for hindering the spread of viruses (Nowzari et al. 2016).

It is well established that network topology is a major factor governing spreading processes (Peng et al. 2020; Cherifi et al. 2019), and it has been shown that real world networks often exhibit small-world properties with scale-free degree distributions, which allows viruses and information to proliferate and cover large parts of such networks quickly (Pastor-Satorras and Vespignani 2001; Wu et al. 2004). One overarching goal of most studies on information diffusion in networks is to predict the efficiency of the underlying spreading process, i.e. how quickly and how pervasively the nodes in a network get infected by a spreading item (information, rumor, virus, etc.). Being referred to as immunisation or attack strategies, they usually examine which nodes exert the most influence on the information diffusion process, i.e., are the most influential spreaders, in order to either immunise or attack them and better control the spreading (Cherifi et al. 2019; Magelinski et al. 2021). In this context, topological properties of nodes (e.g., centrality, community membership) are of interest. Such properties can be discovered by using local information on the node level or by examining the global network structure. A particularly important topological feature which influences the efficiency of the spreading process is the community structure of networks and the resulting properties of nodes (Rajeh et al. 2021; Ghalmane et al. 2019a, b; Kitsak et al. 2010). To this end, Kitsak et al. (2010) showed that the most efficient spreaders within a network are not necessarily the most central nodes (i.e. nodes with the highest degree), but the ones located in densely connected cores of the network indicated by a high *k*-shell index. This insight was accompanied by our recent study on the positive effect of node membership in multiple overlapping and densely connected clusters in a network (Krukowski and Hecking 2021). Here, the general idea is that someone who is member of many different overlapping social groups (workplace, sports club, friendship circles) is better capable of injecting a spreading item into various densely connected regions of the network where it further circulates. Multiple approaches exist to detect such overlapping clusters (Xie et al. 2013). In this paper, we use the link-communities algorithm by Ahn et al. (2010), where resulting clusters are highly interleaved and sometimes even nested. The spreading capability of a node is then modelled as a function of the number of such clusters in which it occurs.

The discovery of such node properties and the resulting immunisation strategies differ in how computationally expensive and how applicable to real-world scenarios they are. Strategies which use typical measures of centrality or the *k*-Shell index of nodes afford knowledge about the full network structure. For scenarios like information diffusion in social media, such knowledge is usually given. However, this is not the case for real-world contacts. Especially in the light of global pandemics, such as the current COVID-19 crisis, it would be desirable if one could approximate the spreading capability of nodes only using information from their immediate neighbourhood, i.e. on the node level. This would greatly improve personalised warning systems, for example based on contact tracing apps, without the need for collecting personal contact information at a central organisation.

Accordingly, this article extends the results of our previous work (Krukowski and Hecking 2021) on properties of the community structure as indicators for the spreading capability of nodes and how well these can be approximated, yet providing a more extensive evaluation particularly with regard to comparison of global and local approximations.

The paper is structured as follows: In the “Background” section, we delineate the background and outline the necessary preliminaries. Related work on this topic will be described in the “Related work” section. Our global and local approaches for identifying influential spreaders in networks are explained in the “Methods” section. Evaluation results on real-world and generated networks are reported in the “Analysis” section and eventually discussed in a broader scope in the “Summary and conclusions” section.

## Background

Spreading processes generally describe a flow of information between actors or members of a network (Karunakaran et al. 2017). Hence, within complex networks, spreading can only happen between adjacent nodes, which makes topological features important factors to consider when attempting to control the spreading process. For immunisation strategies, typically the origin of the diffusion process is of interest, as these so called “seeds” (Comin and Costa 2011) and their properties yield important information from which inferences regarding the efficiency of the spreading process (i.e., their spreading capability) can be drawn. For example, the degree centrality of a node is one such feature, as nodes with high degree centralities naturally have more possibilities to potentially spread information to other nodes (Albert et al. 2000). Thus, these so called “hubs” mark efficient spreaders (Erkol et al. 2019). This is also reflected by the fact that an uneven degree distribution within networks (many hubs) results in more efficient spreading (Barabasi 2016). Apart from hubs, the community structure of graphs is another important factor influencing spreading processes (Rajeh et al. 2021; Ghalmane et al. 2019a, b). Information spreads more easily within highly connected sub-communities (Stegehuis et al. 2016), and similarly, nodes who act as bridges or are members of multiple overlapping communities might also spread information more easily between communities. As such, considering the community structure can improve the prediction of the spreading process and thus the immunisation of relevant nodes (Peng et al. 2020; Ghalmane et al. 2019a).

### Community structure and spreading

In this paper, we focus on this community structure of networks and examine, how the membership of nodes in different overlapping communities might help predict their spreading capability. To this end, we relate this property to other properties of community structure, specifically the *k*-Shell index of nodes.

#### *k*-Shell Index

**Fig. 1:**
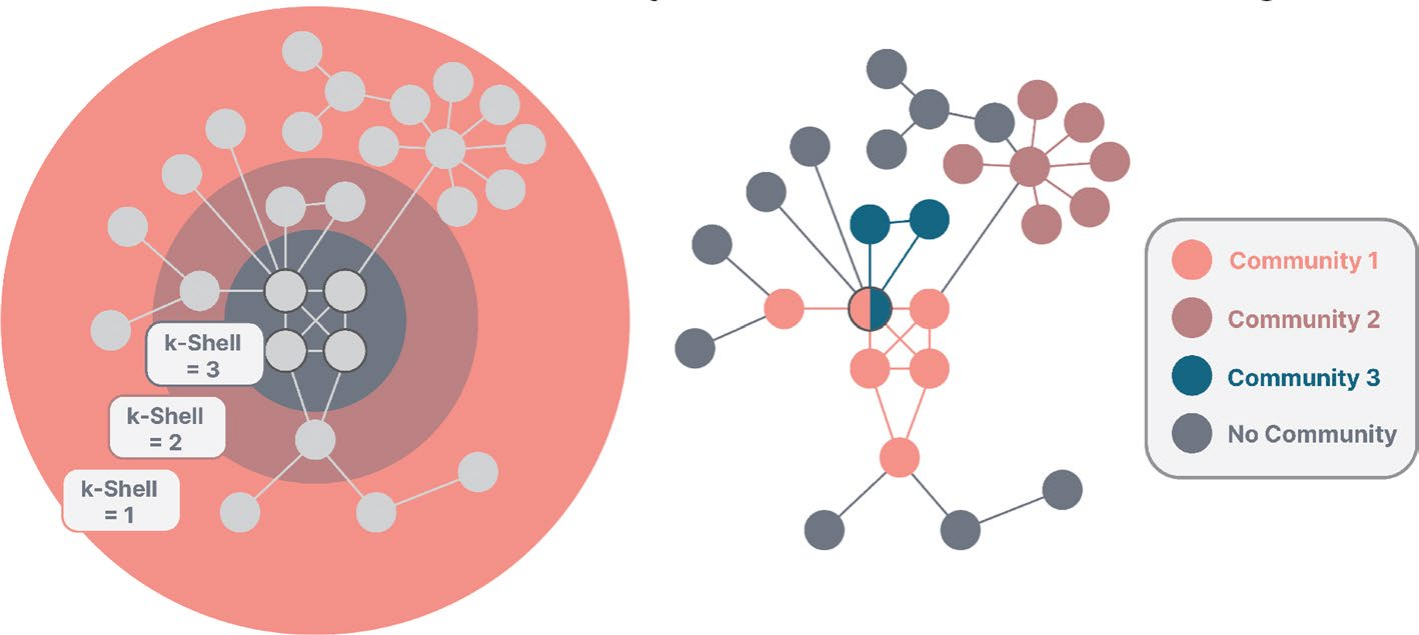
On the left, an example from Kitsak et al. (2010) can be seen. Nodes within the core of the network, i.e. with a high *k*-Shell index, were found to be good spreaders. On the right, the same network is shown, but clustered with the Link Communities approach by Ahn et al. (2010). Nodes with grey borders are nodes which are hypothesized to exhibit a high spreading capability

Within highly cohesive subgraphs, information can spread more easily. A common notion in graph theory to calculate this cohesiveness is to determine the *k*-Core of a network. This* k*-Core (Dorogovtsev et al. 2006) refers to the largest induced subgraph of a network, in which all nodes have at least a *k* connections with others. It can be determined by successively removing nodes from the network with degree smaller *k* until no more nodes can be removed. The *k*-Shell index of a node then indicates the largest *k*-Core it belongs to. Thus, nodes with a shell index of *k* have at least *k* connections to other nodes within their core, meaning that high *k*-Shell values indicate a membership within highly interconnected subgraphs where information can flow easily between all its members (see Fig. 1). It is reasonable to assume then, that a spreading process which starts within those highly interconnected subgraphs is more efficient than when the seed node is simply highly connected (i.e., has a high degree). To this end, Kitsak et al. (2010) could show, that the *k*-Shell index of a node is indeed a superior predictor for its spreading capability as opposed to its degree, specifically by sustaining infections within early stages and helping them reach a critical mass. However, nodes within these *k*-Cores also tend to exhibit multiple community memberships, which yields an additional predictor in comparison to solely taking the node’s *k*-Shell index into account. Additionally, when multiple outbreaks happen, information can spread more easily between different sub-communities, whereas for different cores, the distance between them needs to be taken into account (Kitsak et al. 2010). Thus, another connected, but distinct topological feature which might impact the spreading efficiency is the community membership of nodes.

#### Link communities and influential spreaders

**Fig. 2:**
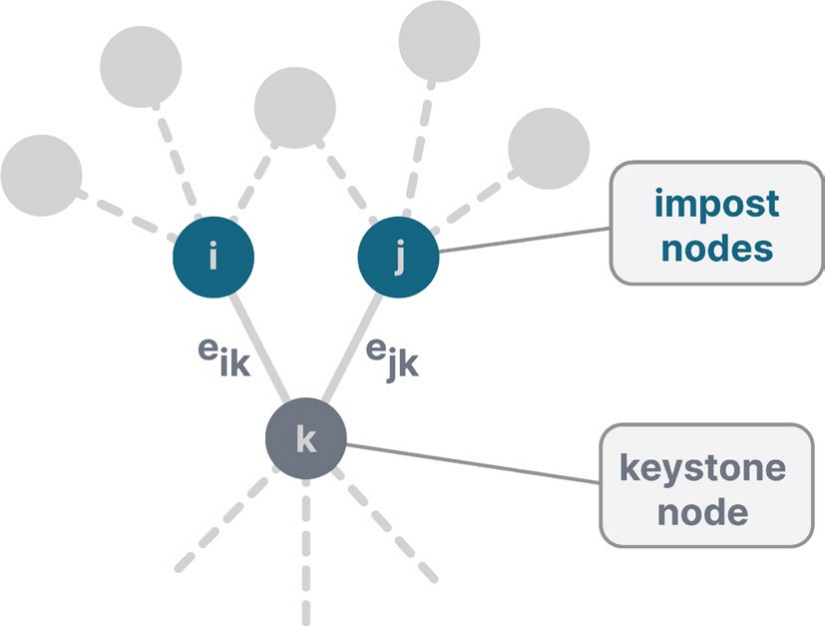
Illustration from Ahn et al. (2010). As can be seen, only the neighbourhood of the impost nodes is taken into account

To examine the community structure of graphs, community detection techniques such as the Louvain method (Blondel et al. 2008) can be used. The Louvain method assigns sub-communities to nodes based on high connections within a community and little connections between different sub-communities, resulting in each node getting assigned a unique sub-community. However, especially nodes which are part of multiple overlapping and nested communities, i.e., connect groups in different social contexts, might be capable spreaders. The underlying assumption is that information, diseases, etc. mainly circulate within densely connected groups. Actors in the overlap of such groups can be infected within one group and inject the spreading processes into several other densely connected groups. Similarly, nodes in overlaps are often neighbours of highly influential nodes (i.e., hubs) within their respective communities, which allows for an even more efficient spreading. In contrast to node partitioning methods such as the one mentioned above, the Link Communities approach by Ahn et al. (2010) was especially designed to uncover communities with pervasive overlaps. Based on the assumption that social groups are better characterised as a set of closely interrelated links instead of closely interconnected nodes, the method partitions the links of a network instead of the set of nodes. As a result, sub-communities can overlap, and single nodes can be members of multiple sub-communities. The procedure of link clustering is described as follows: Edges ($e_ik$ and $e_jk$ ) with a common neighbour k are compared pairwise. Node k is called keystone node, while the other two nodes are called impost nodes (see Fig. 2). It should be noted, that only the neighbours of the impost nodes are taken into account for the calculation, as the neighbours of k (except the impost nodes) are of no interest. To calculate the similarity of the nodes, the similarity criterion *S* (Jaccard- index) is applied (see Eq. 1). The set of the node *i* and its neighbours is denoted as $n + i$.

1 $$\begin{aligned} S(e_ik,e_jk) = \frac{\mid n + (i) \cap n + (j) \mid }{\mid n + (i) \cup n + (j) \mid } \end{aligned}$$

For the above example in Fig. 1, this would result in $S = \frac{1}{4}$ . A dendrogram is then built through single-linkage hierarchical clustering and cut at a certain threshold according to the partition density, which then results in the link communities. From these link communities, the community memberships of the nodes can be derived, and thus each node is assigned a vector of community memberships, from which the actual number of communities it belongs to can be calculated.

In our recent publication (Krukowski and Hecking 2021), we could show that the above hypothesised relationship between the community membership of nodes and their spreading capability does exist, and that nodes, which are members of multiple overlapping sub-communities, do indeed prove to be influential spreaders. Details about the utilisation of the algorithm for detecting influential spreaders will be given in the “Estimating spreading capability using global information” section. Before that, we will shortly discuss related work on spreading processes and the role of the community structure.

## Related work

In recent years, there has been a growing body of research on the role of community structure and how it relates to spreading processes (Cherifi et al. 2019; Tulu et al. 2018). Particularly, the calculation of centrality measures (e.g., degree, betweenness) in a differentiated manner to reflect a node’s local and global influence (Rajeh et al. 2021; Ghalmane et al. 2019b) was shown to lead to a more accurate prediction of efficient spreaders. In this regard, Ghalmane et al. showed that nodes with high local centralities (i.e., high local or within-community influence) seem to be especially capable spreaders in networks with a strong community structure, while those with high global centralities (i.e., high global or inter-community influence) are more efficient in networks with a weaker community structure (Ghalmane et al. 2019b, 2018). Furthermore, network modularity, a major factor representing the community structure of networks, was shown to influence the speed of spreading processes (Peng et al. 2020), and the individual contribution of nodes to the network modularity appears to predict spreading efficiency, as nodes who act as bridges or hubs (and whose deletion would result in a different network modularity) were shown to be especially capable spreaders (modularity vitality, see Magelinski et al. 2021). Thus, many immunisation strategies based on community-structure features of nodes exist (for an overview see Cherifi et al. 2019). These strategies differ with regard to the amount of information about the network structure they need. Community-aware centrality measures, modularity vitality and the *k*-Shell index of nodes presume knowledge of the global network structure, and the resulting immunisation strategies can be characterised as global strategies (Cherifi et al. 2019). In contrast, local immunisation strategies that do not rely on such global information, might be less computationally expensive and more applicable to real-world scenarios (Cherifi et al. 2019). However, even if immunisation occurs on the node level, most algorithms still need information that goes beyound the immediate neighbourhood of nodes. As a typical representative of such local immunisation strategies, the Random-Walk Overlap Selection (RWOS) algorithm requires a pre-specified list of nodes occurring in overlaps between communities, which needs at least partial knowlege about the networks community structure. It performs a random walk on the entire network and counts how often the overlap nodes are encountered. In this regard our approach using a measure of local community centrality described in the next section is fundamentally different, since we aim to find ways how a node can assess its own spreading capability when only the immediate neighbours and the links among them are accessible. This is, for example, the case for real-world contacts collected through contact tracing apps or for users of social networking platforms who can only observe connections between their friends.

## Methods

While there are good indicators that the *k*-Shell index of nodes or link communities work well for identifying influential spreaders in networks (Krukowski and Hecking 2021; Kitsak et al. 2010), the drawback of the required knowledge about the global network structure warrants strategies which allow for an immunisation of relevant nodes irrespective of such knowledge. Especially when considering that for generated or social networks, such knowledge is usually given, it becomes apparent that in real-world scenarios, the complete network with all nodes and their respective links is often unknown - especially in those cases, where the prediction of the individual spreading capability is desired. For example, during the current COVID-19 crisis, a risk-approximation based only on the knowledge of the immediate neighbourhood is both highly desirable and technologically feasible (contact-tracing apps).

Thus, in addition to measures which use global information to approximate spreading capability, we developed a measure which relies only on local information.

### Estimating spreading capability using global information

As described above in the “Link communities and influential spreaders” section, the community membership of nodes in multiple overlapping link communities might be a good indicator for their spreading capability. To this end, we defined a new indicator for the spreading capability of nodes based on the Link Communities algorithm, which is called *global community centrality* ($global\_cc$).

However, due to the nested nature of link communities, a node can be part of many communities that share a large fraction of their nodes so that the number of communities of a node alone may not be sufficient to identify nodes that are able to inject a spreading process in several different densely connected regions of a network. Therefore, the global community centrality of a node *x* participating in *n* link communities $C_1, C_2, \dots , C_n$ is defined as the union nodes in these communities (see Eq. 2).

2 $$\begin{aligned} global\_cc(x) = |\bigcup _{i=1}^n C_i| \end{aligned}$$

This assigns high values to nodes that connect many large communities that do not share many nodes.

### Estimating spreading capability using local information

As mentioned before, a drawback of global methods is that they can only be applied in use-cases where one can view the entire (or large parts) of the network of interest at once.

To estimate the spreading capability of nodes with using local information of their neighbourhood only, we developed an index that approximates global community centrality. The procedure of calculating the *local community centrality* $(local\_{cc})$ of node *x* is at follows: First, an induced subgraph *SG*(*x*) containing all neighbours of *x* is extracted from the entire network. In order to approximate the number of communities in which ego participates, the nodes in *SG* are partitioned using the Louvain Method (Blondel et al. 2008). The rationale behind this is that the number of non-overlapping communities found in the neighbourhood network of *x* correlates with the number of overlapping link communities in which *x* occurs. High values should be assigned to nodes with many well connected local communities in their neighbourhood. The local community centrality of node *x* is given as:

3 $$\begin{aligned} local\_cc(x) = \sum _{c \in C_{SG(x)}} (|E_c| + 1) * |V_c| \end{aligned}$$

$C_{SG(x)}$ is the set of clusters in *SG*(*x*) detected by the Louvaine Method. $E_c$ and $V_c$ denotes the edges and the nodes respectively present in a neighbourhood cluster $c \in C_{SG(x)}$. Note that if there are only isolated nodes (or single-node communities) in the *SG*(*x*), the measure reduces to the degree of *x*. However, if the neighbourhood of *x* can be partitioned into several densely connected clusters, *x* has a good chance to be a good spreader since it possibly is a connector of several communities on the global level.

## Analysis

### Modeling spreading capability

To evaluate the capability of nodes to spread information through the network, we simulated spreading processes according to well-known SIR models (May and Lloyd 2001) (**S**usceptible, **I**nfected, **R**ecovered) using Epidemics on Networks (EoN) Python package (Miller and Ting 2019). In this evaluation the process starts with one initially infected node and all others are susceptible. This node tries to infect its susceptible neighbours and succeeds with a given infection rate (denoted as *b*). After that, it recovers and cannot be infected again. The resulting newly infected nodes then in turn try to infect their neighbours. The process terminates when no new infections occur. For each node, 100 of such simulations were conducted and of those, the average number of infected nodes (total infection), the maximum number of infected nodes across all simulation steps (peak infection), and the number of simulation steps until termination (duration) was measured. These measures of the true spreading capability of nodes are then compared to the spreading capability estimated by the different methods described above.

In the following, the results of the evaluation will be presented. All of the evaluations are calculated for *b* (infection rate) = 0.1. To increase the external and internal validity of our evaluations, we examine both real-world and artificially-generated networks.

### The imprecision function

To quantify the importance of nodes with a high local and global community centrality during the spreading, we calculated an objective measure, namely the *imprecision function*. Similar to Kitsak and colleagues (Kitsak et al. 2010), this function is calculated for each of the relevant measures, and is denoted as, $\epsilon _{degree}{(p)}\dots \epsilon _{CC\_local}{(p)}$. For each subset *p* of nodes (here, *p* refers to a specific percentage of the dataset) with the highest spreading capability (denoted as $\phi _{eff}$) and the highest value according the respective measure (denoted as $\phi _{d} \dots \phi _{cc\_local}$), the average spreading is calculated. Then, the difference in spreading between the *p* nodes with highest values in the respective measure and the most efficient spreaders is calculated. Formally, for $\epsilon _{CC\_local}{(p)}$, the function is defined as follows:

$$\begin{aligned} \epsilon _{CC\_local}{(p)} = 1 - \frac{\phi _{CC\_local}(p)}{\phi _{eff}(p)} \end{aligned}$$

By subtracting the fraction from 1, higher values correspond to more imprecision, and smaller values for $\epsilon$ indicate less imprecision and therefore a better measure.

### Real-world networks

We chose two representatives of real-world networks to evaluate different measures for predicting spreading capabilities of nodes, both representing social media data and physical encounters between people.

**Table 1:** Properties of the two real-world networks

	Facebook	SFHH
Nodes	4093	403
Edges	88234	9565
Diameter	8	4
Avg. total infections (sd)	0.35 (0.32)	0.66 (0.4)
Avg. peak infections (sd)	0.1 (0.09)	0.33 (0.2)
Avg. duration (sd)	8.7 (7.2)	6.25 (3.8)

The first network is a sample of a friendship network from Facebook (McAuley and Leskovec 2012). The dataset was assembled from a survey on social cycles on Facebook and is made up of the union of 10 ego networks of survey participants (connections of ego with all of its neighbours + connections between the neighbours). Therefore, it is well suited for evaluation since it can be assumed that it adequately reflects a situation where a node participates in several overlapping social circles. The *Facebook network* is fully connected and comprises 4093 nodes and 88234 edges.

The data of the second real-world network^1^ was collected during the 2009 SFHH (Société Française d’Hygiène Hospitalière) conference. The 403 nodes represent the participants. Each participant wore RFID (radio-frequency identification) devices for close proximity contact tracking. The edges in the network represent face-to-face encounters between the participants for more than 20 seconds. In the following this network will be referred to as the *SFHH network*. Table 1 summarises the main properties of the network including statistics of the SIR simulations with infection rate 0.1 and 100 runs per node. The duration column refers to time steps of the simulation. It can be seen that in the smaller SFHH network a much larger proportion of nodes get infected more quickly, which is due to the smaller diameter and higher average degree.

**Table 2:** Pearson correlations of the true spreading capability of nodes with the different measures in the Facebook network

	Total infection	Peak infection	Duration
Degree	0.68	0.63	0.68
*k*-shell	0.68	0.8	0.73
global_cc	0.7	0.64	0.7
local_cc	0.2	0.17	0.19

**Table 3:** Pearson correlations of the true spreading capability of nodes with the different measures in the SFHH contact network

	Total infection	Peak infection	Duration
Degree	0.42	0.42	0.4
*k*-shell	0.36	0.37	0.34
global_cc	0.63	0.63	0.61
local_cc	0.51	0.51	0.49

A first observation is that there is a medium correlation between nearly all structural node properties and the spreading capability of nodes, indicating that the true spreading capability is linked to these properties (see Tables 2, 3). The only exception is the correlation of the local community centrality in the Facebook network, which appears to be only small. However, correlations alone do not provide enough insights into the utility of different immunisation strategies. Therefore, the imprecision function described in the “The imprecision function” section was calculated at different values for *p* (i.e. the top fraction of nodes according to the measures and true spreading capability).

**Fig. 3:**
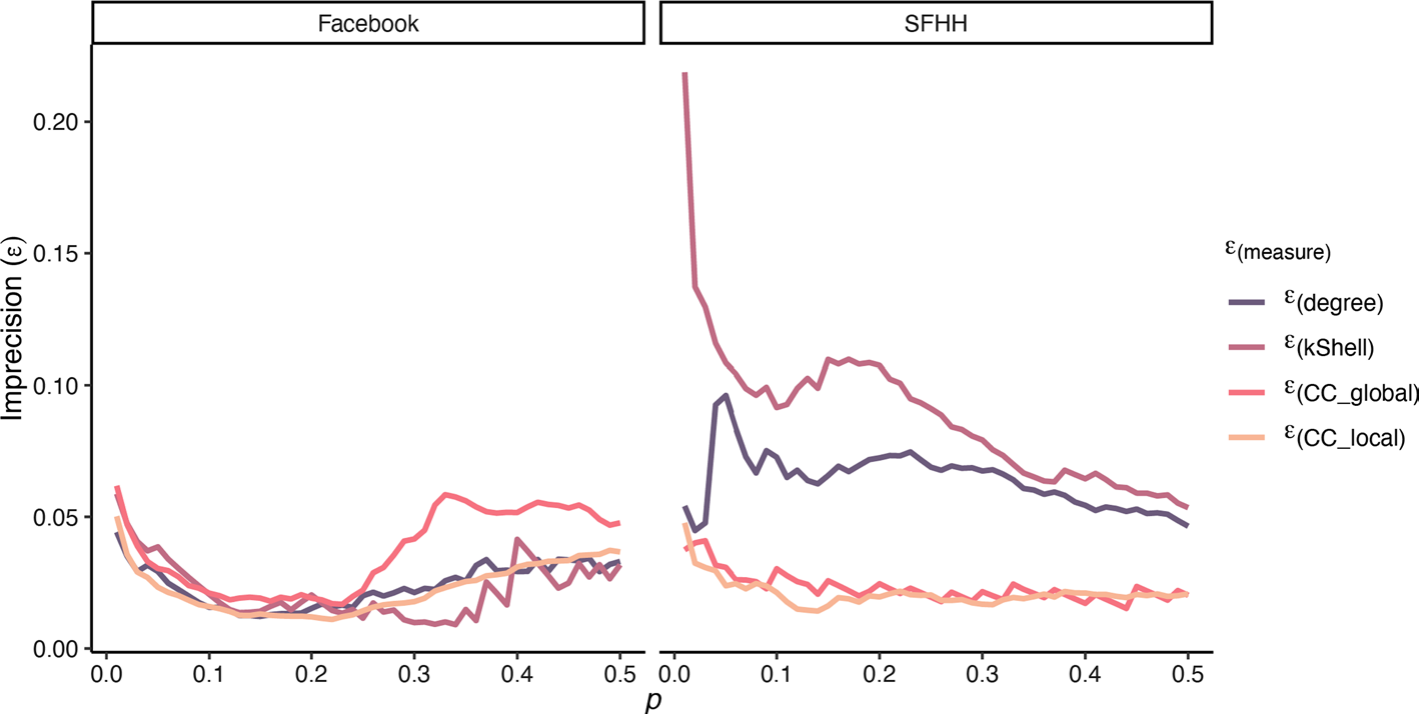
Imprecision of identifying the top *p* fraction of spreaders by different measures over growing *p* with regard to the total infection

**Fig. 4:**
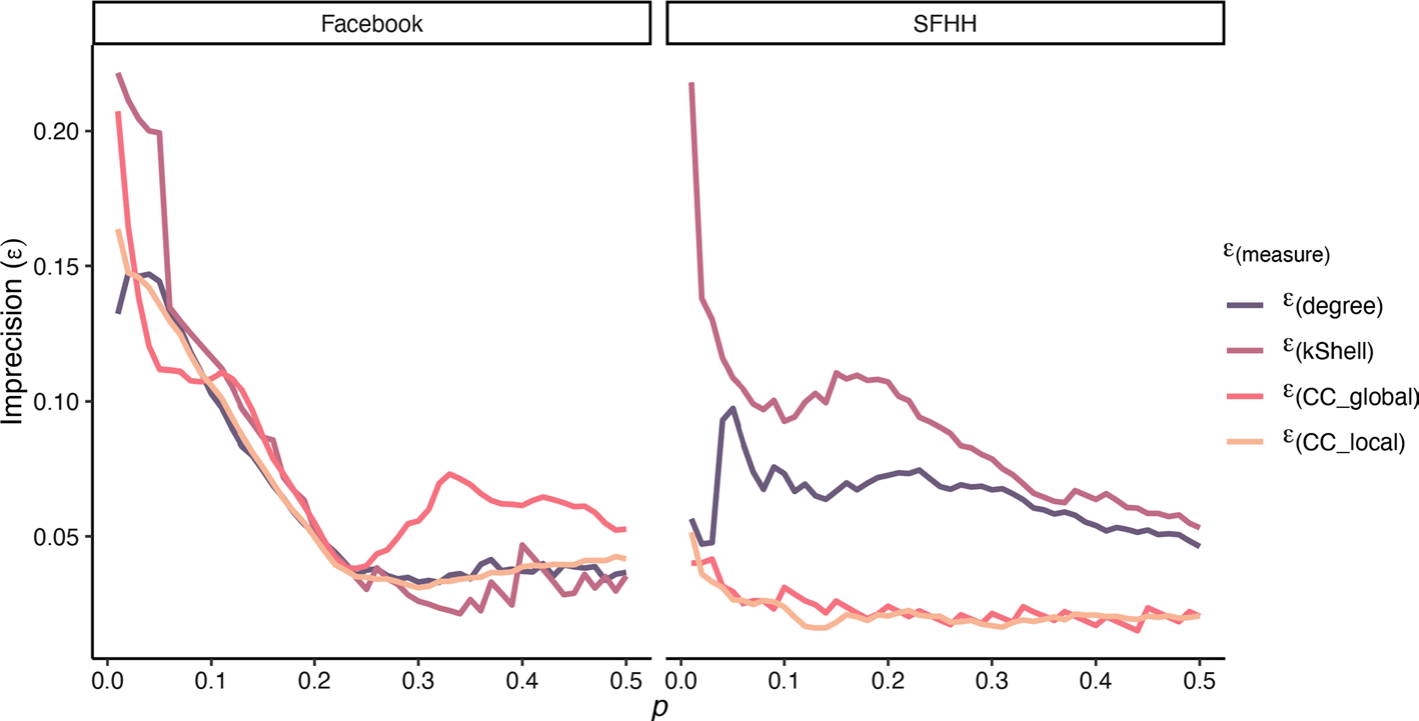
Imprecision of identifying the top *p* fraction of spreaders by different measures over growing *p* with regard to the peak infection

Here, the results for the two real-world networks are mixed concerning the identification of top spreaders in the two networks (see Fig. 3 for the total number of infected nodes and Fig. 4 for the peak infection). For total infection (see Fig. 3), mean imprecision values across all *p* values are 0.02 for degree, 0.02 for *k*-Shell, 0.04 for $global\_cc$ and 0.02 for $local\_cc$ for the Facebook network, while for the SFHH network they are 0.06 for degree, 0.09 for *k*-Shell, 0.02 for $global\_cc$ and 0.02 for $local\_cc$. For peak infection (see Fig. 4), mean imprecision values across all *p* values are 0.06 for degree, 0.07 for *k*-Shell, 0.08 for $global\_cc$ and 0.06 for $local\_cc$ for Facebook, while for SFHH, they are 0.06 for degree, 0.09 for *k*-Shell, 0.02 for $global\_cc$ and 0.02 for $local\_cc$. Thus, judging from the mean imprecision values alone, no clear picture regarding our local and global community centrality measures emerges, although they appear to be comparable to the other measures in identifying top spreaders. Contrary to the findings by Kitsak et al. (2010), the *k*-Shell index is not superior to the degree and other measures for small values of the top fraction of spreaders *p* in our analysed networks. In the Facebook network, however, the differences between the measures are marginal in this regime and the *k*-Shell index appears to be superior for $p > 0.28$. This can be attributed to the smaller variance of the spreading capability of nodes in the Facebook network (see Table 2). Furthermore, in the Facebook network there are only 75 out of 4039 nodes which have a *k*-Shell index smaller than 2, and thus, a large periphery with many star-like structures (i.e., one high degree node is connected to many nodes with low degrees) is not present in the network so that the degree of a node is a good proxy for spreading capability. In contrast to that, in the SFHH network, both the community centrality on the global ($global\_cc$) as well as the local level ($local\_cc$) clearly better identify the top spreaders compared to the degree or *k*-Shell index of nodes. It can be seen in Figs. 3 and 4 that both measures consistently show smaller imprecision values for the majority of *p*-values and hence identify influential spreaders with more precision. Since this network represents a real-world contact network at an event, more nodes emerge who connect different densely connected regions of the network if they move from one group to another.

**Fig. 5:**
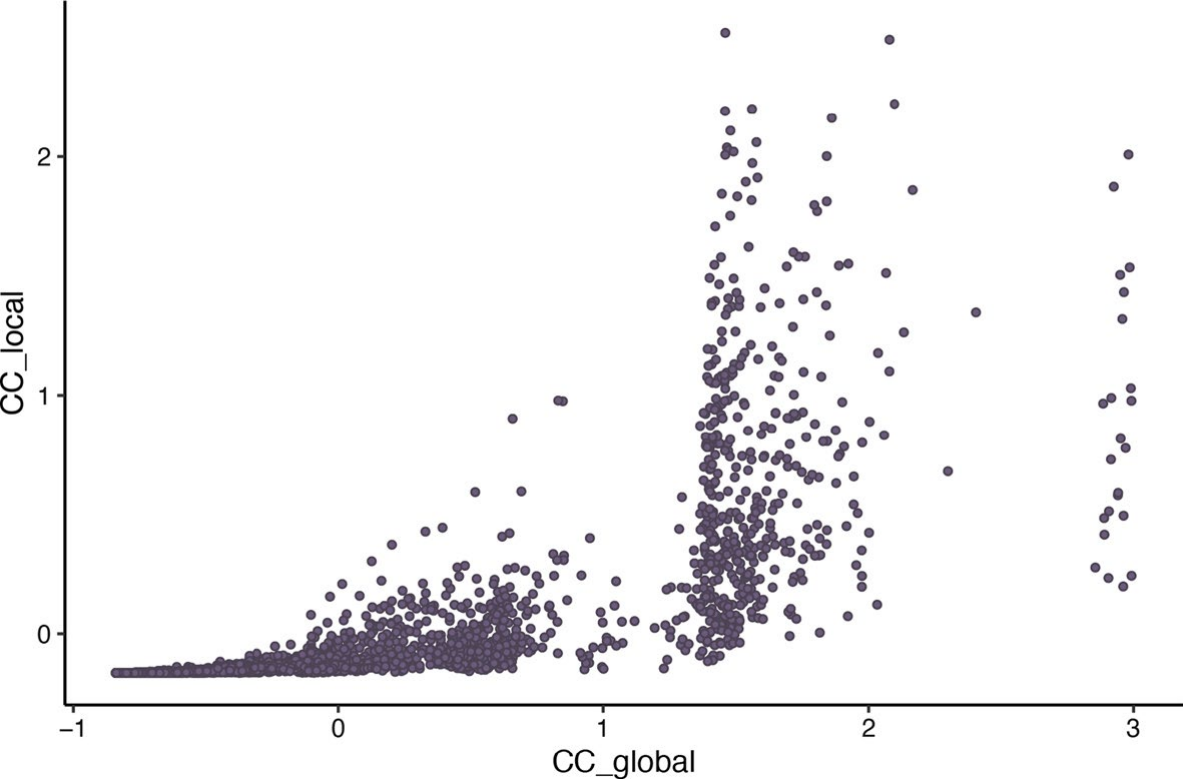
Scatterplot of the z-standardised values of the local and global community centrality for each node. Values deviating > 3 SDs from the mean were excluded from plotting

To additionally analyse how the $local\_cc$ and $global\_cc$ relate to each other, we generated a scatterplot which can be seen in Fig. 5. The plot shows the local and global community centrality for each node in the Facebook network. As one can see, the measures correspond to each other, especially for smaller values, confirming our intuition that the non-overlapping communities found in the neighbourhood network of nodes correlates with the number of overlapping link communities in which they occur.

### Generated networks

From the previous section it becomes clear that measures of identifying top spreaders need a differentiated consideration in relation to properties of the underlying network. To this end, we generated 8 networks of 1000 nodes according to different configurations of the Forest-Fire Model (Leskovec et al. 2007), as it creates networks with typical properties of real-world networks such as heavy-tailed degree distributions and community structure.

The model was configured in the following manner: First a forward burning probability $p_{fb}$ has to be specified. The generator adds one node at a time and it randomly connects to one of the already exiting nodes. For each node *x* that gained a new edge, a random number *r* is sampled from a geometric distribution with mean $p_{fb}/(1-p_{fb})$. The new node then connects to *r* neighbours of *x*. The procedure continues for each newly established connection until termination. For each of such new *forward burning connection*, a new triangle emerges. Consequently, forward burning creates densely connected regions in the network. Since $p_{fb}$ is chosen typically small, forward burning succeeds only for a small fraction of the neighbours of *x* and can propagate in different network regions that are connected by *x*. In this way, pervasively and nested communities emerge in a natural way, which makes this model particularly suited for our analysis. Furthermore, it is known that the Forest Fire model inherently models preferential attachments and creates communities having properties that can be observed in many real-world networks (Leskovec et al. 2009). Depending on the neighbourhood of the node *x* burning of only a few steps creates small and well separated communities while burning with a higher success rate creates large communities that merge with others eventually forming the network core.

We generated networks with a forward-burning probability $p^1_{fb} = 0.05, p^2_{fb} = 0.1, p^3_{fb} = 0.15, \dots , p^8_{fb} = 0.4$. The higher the forward burning probability, the denser the networks. Moreover, the parameter controls the number of dense and overlapping communities in the network, namely smaller and more separated communities for small values of $p_{fb}$, while for large values several communities merge into a giant component (Leskovec et al. 2007).

**Fig. 6:**
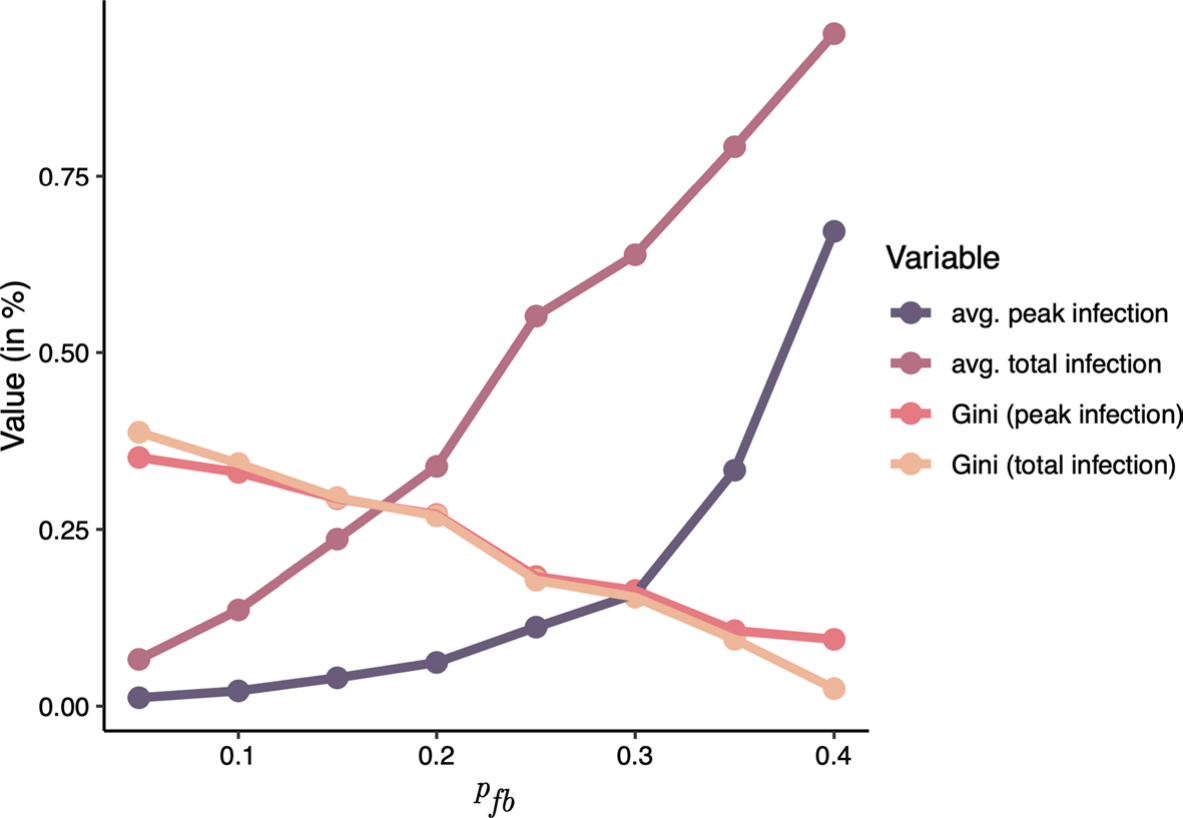
Average infection and gini coefficient for the two infection measures (peak infection and total infection)

As can be seen in Fig. 6, the average infection rate of the nodes increases, as higher forward burning probabilities are chosen for the generation of the networks. This can on one hand be attributed to increasing density of the networks, but also to the number of emerging overlapping communities.

**Fig. 7:**
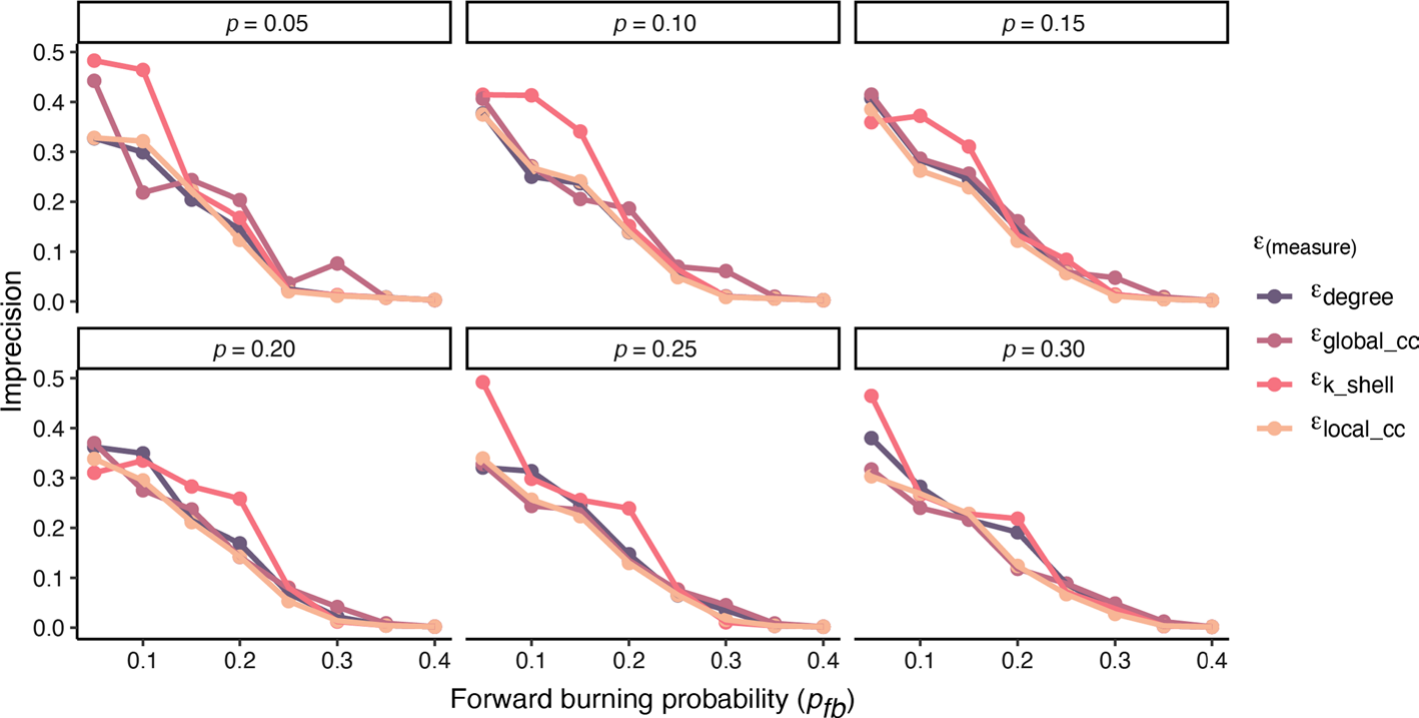
Imprecision of estimating the infection of the *p* top nodes. Values on the x-axis correspond to the $p_{fb}$ value used to generate our 8 networks

By plotting the imprecision function of the top *p* fraction of spreaders selected by the different measures for both total infection and peak infection (see Fig. 7), one can see that the imprecision generally decreases for networks generated with growing forward burning probability. This can clearly attributed to the fact that with large values for $p_{fb}$ the networks are denser and several communities merge into a giant component during network evolution as described above. In such networks, almost every node is a good spreader and all measures perform similar. Across all *p* values, mean imprecision values are 0.14 for degree, 0.17 for *k*-Shell, .15 for $global\_cc$ and .13 for $local\_cc$. However, for networks created with smaller forward burning probability $p_{fb} \le 0.2$ larger differences in the imprecision can be observed (0.26 for degree, 0.31 for *k*-Shell, 0.26 for $global\_cc$ and 0.24 for $local\_cc$) showing that the local and global community centrality are slightly superior to the degree and definitely to the *k*-shell index for identifying good spreaders in such networks. For those generated with forward probability $p_{fb} > 0.2$ however, differences between the measures become marginal. This is most probably due to the fact that these networks are quite densely connected and consequently, every node has good spreading capability.

To exemplify this, Fig. 6 shows the average infection rate for each of the generated networks, along with the normalised Gini coefficient (Dorfman 1979), which was calculated for the spreading capability of nodes (measured by total and peak infection). The normalised Gini coefficient measures the skewness of a distribution (or statistical dispersion). It was originally developed for measuring the wealth inequality of a population. It is 1 if one person owns all and 0 if the wealth status is uniformly distributed over a population. The same can also be applied for the total number of infections for a SIR spreading process started at particular nodes. It can be seen that nodes with significantly higher spreading capability compared the the majority only emerge for lower values of $p_{fb}$, which explains the little deviation between influence measures in networks generated with $p_{fb} > 0.2$, while for the other networks, almost all nodes are good spreaders.

## Summary and conclusions

In this paper, we intended to complement recent findings (Krukowski and Hecking 2021) regarding spreading processes within complex networks and the immunisation of nodes using topological features, specifically the community structure. In doing so, we approach two goals: Firstly, extend our understanding of our recently introduced measure of community centrality ($global\_cc$), and secondly, develop a new measure to predict the efficiency of the spreading process which can be used without knowledge of the global network structure ($local\_cc$). While for $global\_cc$, the community centrality is calculated using link clustering (Ahn et al. 2010; Krukowski and Hecking 2021), for the $local\_cc$, an induced subgraph for every node of the network is created (i.e., ego-network), which is then clustered using the Louvain-method (Blondel et al. 2008). The assumption is that if the neighbourhood of a node *x* can be clustered into several densely connected sub-communities, this is an indicator that *x* connects different social cycles. From the resulting number of communities, its local community centrality is assigned. We showed that this assumption is justifiable by considering the correlation between the local and global community centrality. To approach the goals of this paper, we examined both measures in relation to already established measures used to identify influential spreaders (degree, *k*-Shell). Generally, our results confirm the comparability of the $global\_cc$ measure to the other measures in predicting a node’s spreading capability. The same applies to the newly introduced $local\_cc$ measure, which even performs better than the $global\_cc$ in both our examined real-world networks as well as artificially generated ones. The calculated correlation coefficients show a comparable correlation for all of the examined measures with the spreading capability, except for the $local\_cc$ in the Facebook network. To extend this finding by objectively comparing the respective top-spreaders, we calculated the imprecision function for the *p* top spreaders selected by different measures. For the examined real-world networks, it shows that local measures appear to be suitable predictors for small values of *p* in the Facebook network, while they are generally more accurate in the SFHH network, irrespective of the *p* value (i.e., top % of spreaders). This applies to both peak infection and total infection. However, for bigger values of *p* (top fraction of spreaders selected by a measure) in the Facebook network, the *k*-Shell index outperforms the other measures, although this might be attributable to the absence of star-like structures and real-world properties.

To increase external validity, we additionally analysed 8 artificially generated networks, with the generation parameter $p_{fb}$, which leads to more community-structure and heavy-tailed degree distributions as it increases. Here, along with decreasing imprecision for higher $p_{fb}$ values (i.e., more community structure), it shows that the $local\_cc$ even outperforms the $global\_cc$ - for nearly all of the 8 generated networks. In conclusion, the evaluations showed two things. Firstly, they deepened our understanding regarding topological features and their influence on spreading processes by showing that the $global\_cc$ measure is comparable to the others in our examined real-world networks. As such, it provides further evidence for the influence of community structure on spreading processes, and it confirms the importance community overlaps for spreading processes: Nodes who are in such overlaps not only appear to be more likely in the vicinity of hubs (Ghalmane et al. 2020), they might be well connected to other nodes in the sub-community (Yang and Leskovec 2012), allowing for an efficient spreading to other parts of the network. Our results add evidence to this. Secondly, they show that for a good approximation, knowledge of the global network structure is not necessarily needed. Instead, using the $global\_cc$ measure, spreading capability can be equally well predicted. This confirms related research on this topic, which showed the effectiveness of using local immunisation strategies that do not rely on global network structure (Kumar et al. 2018; Taghavian et al. 2017). It also shows that local information on the direct neighbourhood of nodes corresponds to aspects of the global community structure of networks (see Kudelka et al. 2019). In addition to that, as opposed to other common local immunisation strategies, our measure does not need any pre-calculated or ground-truth knowledge of the community structure, and instead infers such information using local information only, making it computationally more efficient as well as more applicable to real-world scenarios.

The introduction of such a measure is important: While findings regarding topological features and their relation to spreading efficiency are of high theoretical relevance, they lose practical relevance in real-world settings where knowledge of the global network structure is rarely given. Accordingly, topological features such as the *k*-Shell index, modularity or global community centrality offer little inferential value for individuals who want to assess their spreading capability but have knowledge about their immediate local network only. Yet especially in situations like the current COVID-19 crisis, such local approximations of the global spreading capability of nodes could prove highly useful in personalised risk management (e.g. in contact tracing apps) for preventing infections.

## Limitations and future works

The presented study has certain limitations that need to be considered in follow-up works. The results on the Facebook and SFHH networks presented in the “Real-world networks” section compared to the evaluation on generated networks in the “Generated networks” section give good indications under which conditions each measure for identifying top spreaders is most appropriate. Although the SFHH network represents an epidemiologically relevant network, more experiments on additional networks from different domains and with different topologies and sizes are necessary to further consolidate under which conditions each measure is most appropriate. In this regard, making use of voluntary data donations about real-world contacts as they become increasingly available constitute a promising direction. For the evaluation on artificially generated networks, in future works we aim to develop and use new models that allow for a better control for the emergence of overlapping community structures than the used forest-fire model, e.g., the LFR (Lancichinetti–Fortunato–Radicchi) model (Lancichinetti et al. 2008). This would allow to investigate the proposed methods in more detail.

## Data Availability

The datasets used and analysed during the current study are available from the corresponding author on reasonable request.

## Abbreviations

EoN: Epidemics on Networks python package
Global_cc: Global community centrality
LFR: Lancichinetti–Fortunato–Radicchi
Local_cc: Local community centrality
RFID: Radio-frequency identification
RWOS: Random-walk overlap selection
SFHH: Société Française d’Hygiène Hospitalière conference
SIR: Susceptible, infected, recovered

## Footnotes

1 http://www.sociopatterns.org/datasets/sfhh-conference-data-set/.
